# The Establishment of a Sheep Embryo Genomic Selection System

**DOI:** 10.3390/ijms26199738

**Published:** 2025-10-07

**Authors:** Yubing Wang, Hao Qin, Ke Li, Jia Hao, Xingyuan Liu, Dayong Chen, Lei Cheng, Huijie He, Riga Wu, Yingjie Wu, Yinjuan Wang, Min Guo, Qin Li, Lei An, Jianhui Tian, Hongbing Han, Guangyin Xi

**Affiliations:** 1 Laboratory of Animal Genetics, Breeding and Reproduction of the Ministry of Agriculture and Rural Affairs, Frontiers Science Center for Molecular Design Breeding (MOE), College of Animal Science and Technology, China Agricultural University, Beijing 100193, China; wangyubing911@163.com (Y.W.); haoq@cau.edu.cn (H.Q.); like5726@outlook.com (K.L.); 17630751829@163.com (J.H.); 19806395675@163.com (X.L.); wuyingjie@cau.edu.cn (Y.W.); wangyinjuan@cau.edu.cn (Y.W.); guominyzy@163.com (M.G.); crystal_stefer@126.com (Q.L.); anleim@cau.edu.cn (L.A.); tianjh@cau.edu.cn (J.T.); 2 Key Laboratory of Sheep Genetic Breeding and Reproduction Technology, Ministry of Agriculture and Rural Affairs, Ulanqab 011800, China; chendayong81@126.com (D.C.); cl344713520@163.com (L.C.); 15904749394@163.com (H.H.); wuriga000@126.com (R.W.)

**Keywords:** sheep, embryo genomic selection, embryo biopsy, whole-genome amplification, chip-based genotyping, in vitro embryo production, animal breeding

## Abstract

Embryo genomic selection (EGS) is a contemporary breeding strategy that combines genomic selection (GS) methodology with embryo biotechnology. By conducting genotyping and genomic prediction at the pre-implantation stage, embryos with superior breeding value can be identified for transfer, markedly increasing breeding efficiency while reducing the uncertainty and temporal expenditure associated with conventional GS. This study establishes a reliable embryo biopsy-based GS pipeline for sheep, incorporating optimized whole-genome amplification and microcell genotyping techniques. We developed a high-efficiency in vitro sheep embryo production platform compatible with embryo biopsy. Systematic comparison of Multiple Displacement Amplification (MDA) and Multiple Annealing and Looping Based Amplification Cycles (MALBAC) whole-genome amplification systems yielded high-quality genotypes from biopsy samples of embryos containing as few as 10 cells. Imputation using 10× whole-genome sequencing data significantly increased both genotype call rates and accuracy. High concordance was observed between embryo and lamb genotypes, and genomic estimated breeding values (GEBVs) for key growth traits exhibited strong correlations (R^2^: 0.91–0.98). This system enables accurate preimplantation genomic evaluation and provides an efficient strategy to accelerate genetic improvement in sheep breeding programs.

## 1. Introduction

GS has transformed animal breeding by enabling early prediction of genetic merit using genome-wide marker profiles before phenotypes become available [1]. By deriving marker effects from a reference population with both genotype and phenotype data, GEBVs can be calculated for selection candidates irrespective of age or performance records [2,3,4]. This approach has markedly accelerated genetic progress, especially for low-heritability and sex-limited traits [5,6,7,8,9].

A persistent bottleneck of conventional GS remains the delay between birth and genotyping, which constrains further compression in generation intervals [10,11,12]. Embryo genomic selection, the genotyping and selection of embryos before transfer, emerges as a powerful solution to this bottleneck [13]. By biopsying a few cells from preimplantation embryos, generating reliable genotypes, and estimating GEBVs prior to implantation, this strategy allows maximal selection intensity and minimizes costs associated with carrying low genetic merit pregnancies. This technique enhances precision in livestock breeding, improving both quality and production efficiency. This strategy offers a compelling, next-generation solution for accelerating genetic improvement in ruminant livestock.

Substantial progress has been made in implementing embryonic GS in cattle [14,15,16,17,18,19,20]. Successful integration of blastocyst biopsy, low-input DNA sequencing, and advanced imputation methods has enabled accurate GEBV prediction and viable pregnancy rates. In sheep, however, genomic selection is still overwhelmingly post-natal; embryo-based GS remains nascent, hindered by sub-optimal in vitro embryo production (IVEP), poorly defined biopsy and cryopreservation windows, and the absence of a standardized whole-genome amplification (WGA)-imputation platform tailored to ovine embryo biopsies. The present study was therefore designed to establish and validate a complete pipeline for genomic selection in ovine embryos.

## 2. Results

### 2.1. Sheep Embryo Genomic Selection Breeding System

**Figure 1:**
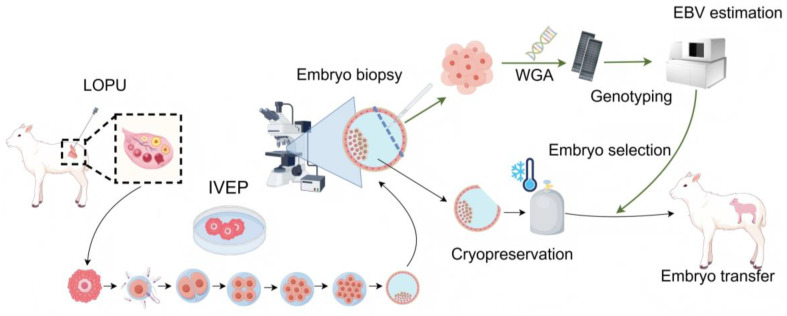
Sheep embryo genomic selection breeding workflow. LOPU, laparoscopic ovum pick-up; IVEP, in vitro embryo production; WGA, whole-genome amplification; GEBV, genomic estimated breeding value. The figure was generated using Figdraw (www.figdraw.com accessed on 28 September 2025).

Figure 1 depicts the schematic workflow for genomic selection in ovine embryos. Donor ewes were subjected to ovarian stimulation using exogenous gonadotropins, followed by laparoscopic ovum pick-up (LOPU) under surgical conditions. The retrieved oocytes were transferred to an in vitro fertilization (IVF) laboratory for maturation, fertilization, and culture to the blastocyst stage. Embryo biopsy was performed at this stage, and the biopsied samples were subjected to whole-genome amplification. The amplified DNA was genotyped using an SNP chip, and the resulting data were analyzed following genotype imputation. Finally, GEBVs were calculated to facilitate the selection of high-quality embryos for transfer.

### 2.2. In Vitro Sheep Embryo Production and Embryo Biopsy

**Figure 2:**
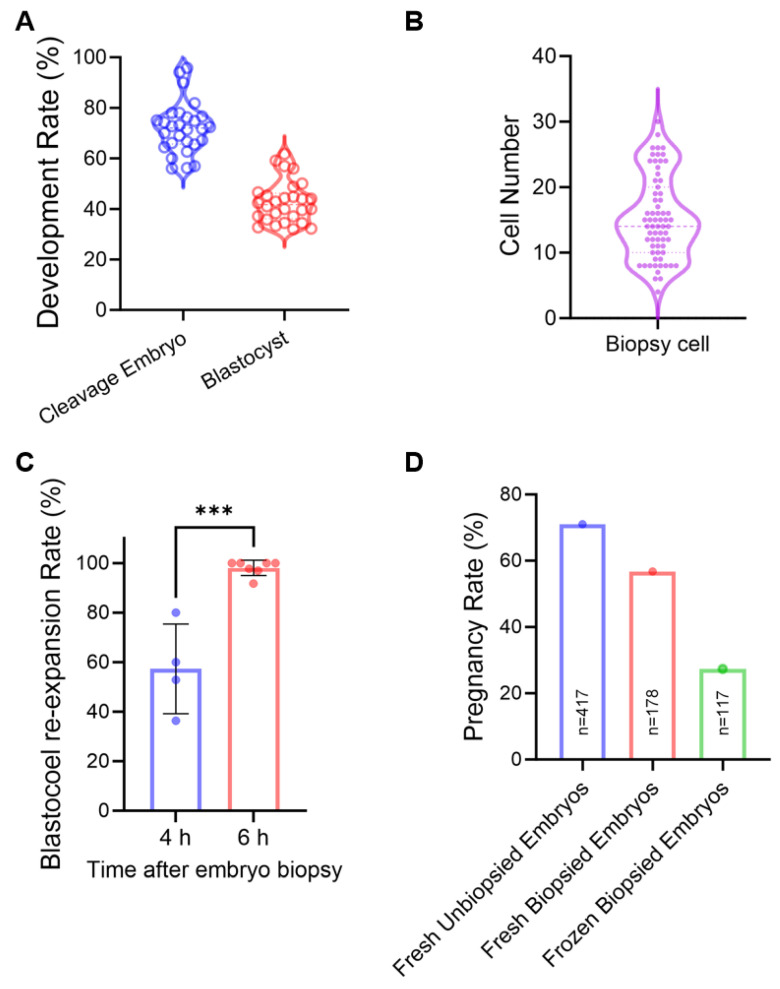
In vitro sheep embryo production and embryo biopsy. (**A**) Cleavage and blastocyst rates of sheep embryos produced under our IVP platform. (**B**) Distribution of cell numbers in biopsied sheep embryo samples. (**C**) Blastocoel re-expansion rate after biopsy at 4 h and 6 h. (**D**) Pregnancy rates following the transfer of fresh unbiopsied embryos, fresh biopsied embryos, and frozen biopsied embryos. Data are presented as mean ± SEM. *** *p* < 0.001.

Viable embryo biopsy procedures were first established and validated based on our sheep IVEP platform. Oocytes were collected via LOPU, followed by in vitro maturation (IVM), IVF, and culture to the D6 blastocyst stage. The platform achieved a cleavage rate of 72.03 ± 1.92% and a blastocyst formation rate of 42.68 ± 1.56%, efficiencies fully sufficient to supply the numbers required for routine embryo biopsy (Figure 2A). Biopsy was performed using a manual microblade method, yielding approximately 15.30 ± 0.77 cells per sample (Figure 2B). Importantly, 98.04% of biopsied embryos successfully re-expanded within 6 h, indicating minimal impact on embryo viability (Figure 2C). However, when biopsy was coupled with vitrification, the pregnancy rate dropped to 27% after transfer, compared to 71% for fresh unbiopsied embryos and 57% for fresh biopsied embryos (Figure 2D), highlighting a significant adverse effect of the cryopreservation procedures on biopsied embryo viability. Nonetheless, these results confirm the technical feasibility of the approach while underscoring the need for further optimization.

### 2.3. Effects of Biopsied Cell Numbers and Amplification Methods on SNP Genotyping

**Figure 3:**
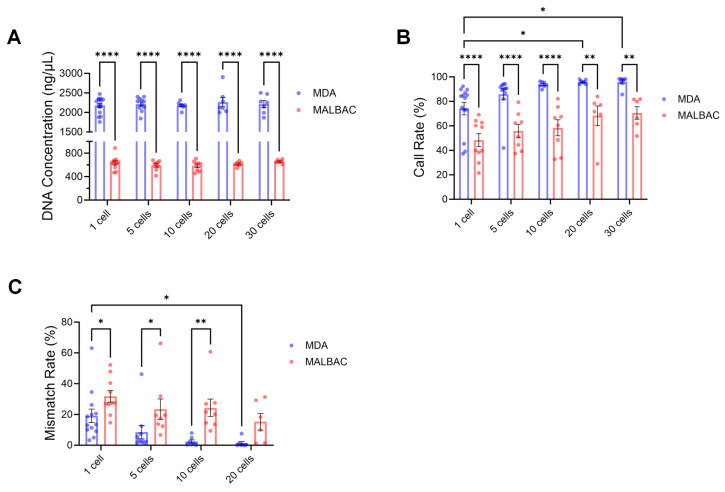
Effects of Biopsied Cell Numbers and Amplification Methods on SNP genotyping. (**A**) DNA concentration of amplified products from biopsy samples with varying cell numbers using MDA and MALBAC whole-genome amplification methods. (**B**) Chip-based genotyping call rates. (**C**) Genotyping mismatch rates. Data are presented as mean ± SEM. * *p* < 0.05, ** *p* < 0.01, **** *p* < 0.0001. Differences not marked with asterisks are not significant (*p* > 0.05).

To ensure accurate genotyping from minute cell samples, the chip-based genotyping protocol was systematically optimized. We compared MALBAC and MDA whole-genome amplification methods. MDA demonstrated superior performance across all evaluated metrics. It consistently yielded higher DNA concentrations, exceeding 2000 ng/μL across varying cell number, whereas MALBAC produced an average of only approximately 600 ng/μL, indicating that MDA offers more efficient and stable amplification in terms of product yield (Figure 3A). MDA achieved higher chip call rate across all cell number, with averages of 74.14 ± 5.14% (1 cell), 85.82 ± 4.52% (5 cells), 93.71 ± 0.90% (10 cells), 95.97 ± 0.63% (20 cells), and 95.91 ± 1.76% (30 cells) (Figure 3B). Furthermore, MDA exhibited lower genotyping mismatch rates compared to MALBAC when evaluated against 30 cells reference samples, with mismatch rates of 12.25 ± 3.02% (1 cell), 4.31 ± 1.22% (5 cells), 2.40 ± 1.11% (10 cells), and 1.39 ± 1.04% (20 cells) (Figure 3C). Based on comprehensive performance evaluation, MDA method is recommended for genotyping sheep blastocysts, with a minimum biopsy requirement of 10 cells to ensure reliability.

### 2.4. Optimization of Biopsy Cell Amplification System

**Figure 4:**
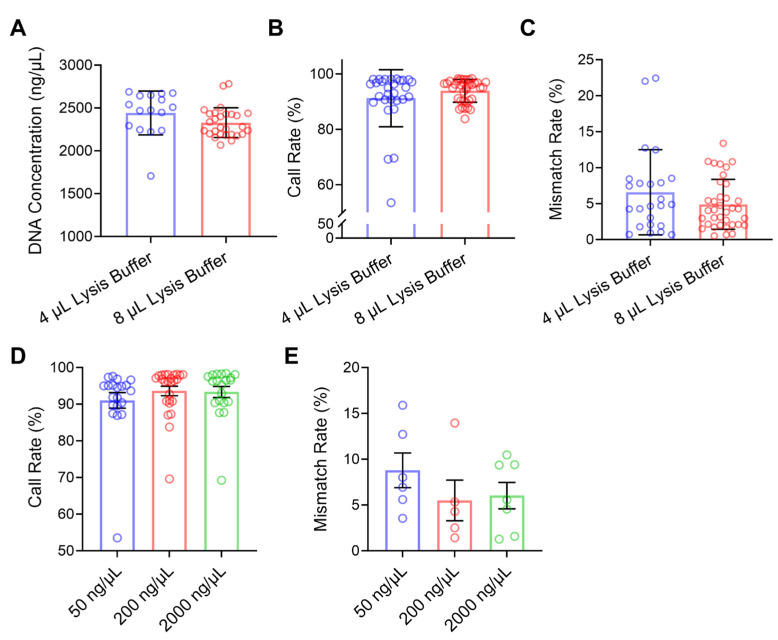
Effects of lysis buffer volume and DNA concentration on chip detection performance. (**A**) DNA concentration obtained using different lysis buffer volumes in the MDA-based WGA system. (**B**) Chip-based genotyping call rate under different lysis buffer volumes. (**C**) Genotyping mismatch rate under different lysis buffer volumes. (**D**) Chip call rate at different DNA loading concentrations. (**E**) Genotyping mismatch rate at different DNA loading concentrations. Data are presented as mean ± SEM.

Next, we investigated the effects of cell lysis buffer volume and DNA concentration on SNP genotyping of biopsied embryo cells. Although lysis buffer volume did not significantly affect amplification efficiency (Figure 4A), call rate, or genotyping accuracy (*p* > 0.05) under the MDA method, practical enhancements were evident. Using 8 μL of lysis buffer resulted in the highest call rate (93.9 ± 0.69% vs. 91.24 ± 1.91% with 4 μL) and the lowest mismatch rate (4.89 ± 0.58% vs. 6.58 ± 1.21% with 4 μL) (Figure 4B,C). Additionally, 8 μL lysis buffer improved the reproducibility of genotyping outcomes, reducing the coefficients of variation (CV) for DNA concentration, call rate, and mismatch rate from 10.48% to 7.46%, 11.28% to 4.38%, and 90.10% to 70.86%, respectively (Figure 4A–C). Thus, 8 μL lysis buffer is recommended for sheep embryo biopsy samples to enhance chip-based genotyping reliability and stability. Furthermore, evaluation of DNA concentration revealed that both 200 ng/μL and 2000 ng/μL yielded higher call rates (93.59 ± 1.30% and 93.33 ± 1.51%, respectively) and lower mismatch rates (5.50 ± 2.22% and 6.03 ± 1.44%, respectively) compared to the 50 ng/μL group (Figure 4D,E). We therefore recommend using at least 200 ng/μL of DNA for chip loading to ensure optimal genotyping performance.

### 2.5. High Genotyping Quality and Consistency Between Embryo Biopsy and Offspring Samples

**Figure 5:**
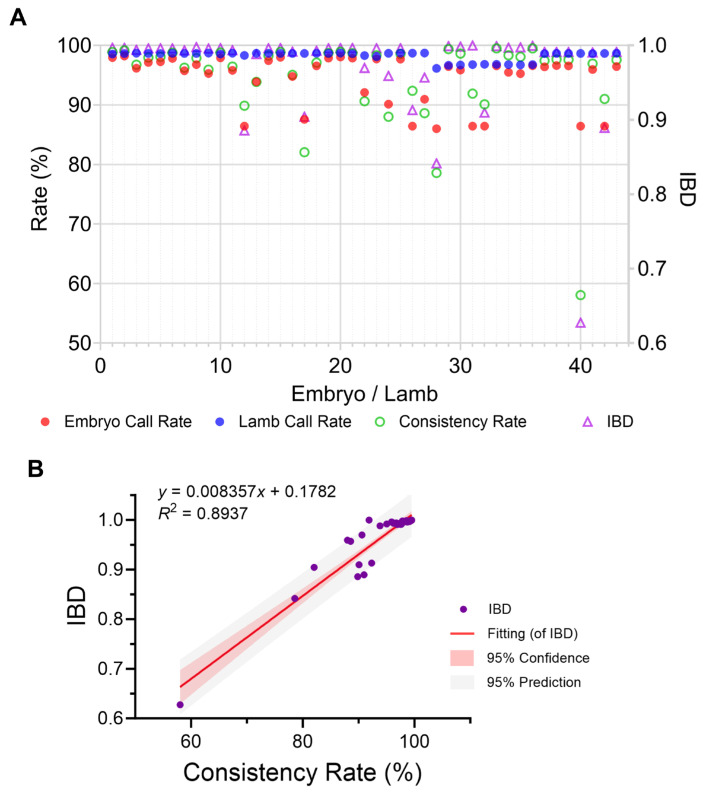
High genotyping quality and consistency between embryo biopsy and offspring samples. (**A**) Call rate, consistency rate and IBD score of genotyping between embryos and lambs. (**B**) Linear fitting of consistency rate of genotyping and IBD score.

The accuracy and feasibility of embryo-based genomic selection were strongly supported by the high genotyping quality and concordance between biopsies and offspring. Embryo biopsy samples and corresponding offspring lambs both exhibited high call rates (86.01% to 98.21% and 96.13% to 98.72%, respectively), indicating robust performance of the biopsy and genotyping workflow. Furthermore, a high genotype concordance was observed between embryonic biopsies and matched postnatal samples, with consistency rates ranging from 58.05% to 99.50% (mean = 94.72%) (Figure 5A). Identity-by-Descent (IBD) scores between pairs ranged from 0.63 to 1.00 (mean = 0.97) (Figure 5A). A strong linear correlation was identified between genotype consistency rate and IBD (R^2^ = 0.89; Figure 5B), underscoring that both genotypic and genealogical evidence converge to support sample identity. These results collectively confirm the accuracy and reliability of sample tracking and genotype data throughout the entire process.

### 2.6. Improved Genotyping Quality Through 10× Whole Genome Resequencing Imputation

**Figure 6:**
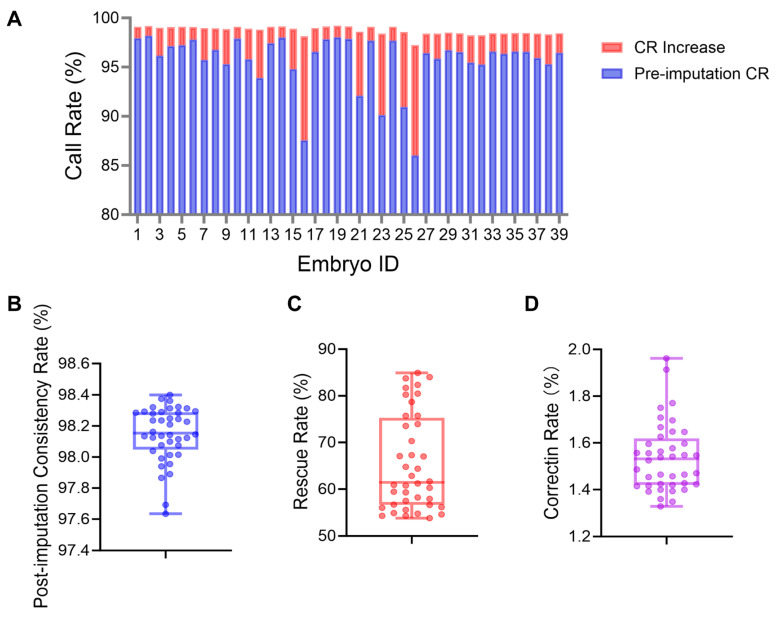
Evaluation of genotype imputation efficiency using 10× whole genome resequencing data. (**A**) Call rate before and after imputation and the extent of call rate improvement. (**B**) Accuracy of imputed genotypes. (**C**) Rescue rate of successfully imputed genotypes. (**D**) Correction rate of imputed genotypes. Data are presented as mean ± SEM. CR: call rate.

Genotype imputation using 10× whole genome resequencing data substantially enhanced the quality of the original chip data. Following imputation, the call rates improved significantly across all samples, rising from a range of 86.01–98.21% to 97.61–98.40%, with individual sample improvements ranging from 0.96 to 11.23 percentage points (Figure 6A). Detailed evaluation confirmed the high efficiency of the imputation procedure, demonstrating excellent final genotype concordance (97.63–98.40%), an effective rescue of missing genotypes (rescue rate: 53.79–84.92%), and a high correction rate for erroneous genotype calls (ranging from 1.33% to 1.96%) (Figure 6B–D).

### 2.7. High Concordance in GEBVs Between Embryos and Offspring

**Figure 7:**
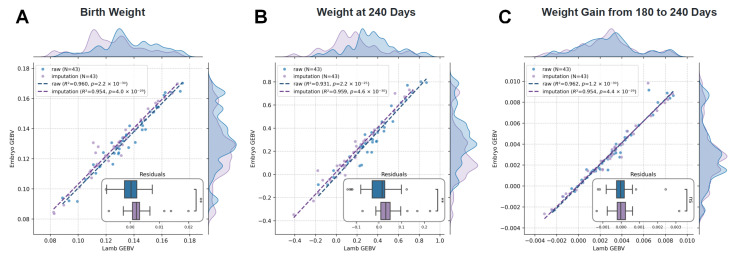
Embryo-lamb GEBV regression-residual composite plot: linear regression of GEBVs derived from embryo genotypes (*y*-axis) against lamb GEBVs (*x*-axis) is presented for both raw (blue) and imputed (gray purple) genotypes (*n* = 43). Three representative traits shown: (**A**) birth weight, (**B**) weight at 240 days, and (**C**) weight gain from 180 to 240 days. The lower panel shows corresponding residuals. Dashed lines represent fitted regressions. The random scattering of residuals around zero across the GEBV range confirms high concordance between embryo and lamb predictions and indicates negligible bias introduced by imputation.

Finally, we evaluated the correlation of GEBVs for eight key growth traits between embryos and lambs, both before and after genotype imputation. The GEBVs derived from embryo DNA showed strong concordance with those from lamb DNA, with R^2^ values ranging from 0.91 to 0.98 using raw data and from 0.94 to 0.98 using imputed data (Figure 7 and Figure S1). The improved consistency after imputation indicates that filling missing genotypes enhanced the accuracy of GEBV prediction for most traits. These results confirm that biopsied embryo genotyping, when combined with imputation, enables reliable early prediction of breeding values. For large-scale applications, considering breeding costs, the accuracy of GEBVs prediction using SNP chip data alone already meets practical requirements, supporting the feasibility of implementing embryo genomic selection system.

## 3. Discussion

Embryo genomic selection, based on SNP genotypes, is gaining traction as a powerful strategy to accelerate genetic gain in cattle [14,16,17,21]. By enabling selection at the preimplantation embryonic stage, it intensifies selection pressure while circumventing the substantial costs associated with gestating and rearing genetically inferior individuals. Although its efficacy has been demonstrated in several dairy cattle populations [18,20,22,23,24]. Here, building on our validated high-efficiency in vitro production platform for ovine embryos, we first optimised and stabilised a manual blastocyst biopsy protocol and a downstream pipeline for micro-DNA whole-genome amplification and SNP genotyping of biopsied cells. We then quantified the empirical correlation between GEBVs derived from trophectoderm biopsies and the GEBVs of the corresponding lambs born after embryo transfer.

Obtaining a sufficient number of embryos is a fundamental prerequisite for implementing and disseminating embryo genomic selection technology, particularly in ruminants, which are predominantly monotocous species. Over the past decades, refinements in livestock IVEP have provided a practical solution to this challenge. In the present study, exogenous gonadotropin protocols reliably induced prolific follicular growth in ewes, yielding 20–22 oocytes per donor at a single ovum pick-up. These oocytes generated 7–9 transferable blastocysts, equating to an IVP efficiency of 40%. Although this output underpins large-scale embryo genomic selection in sheep, it remains markedly below the 70–90% blastocyst rate achieved in vivo [25,26]. The disparity is largely attributed to suboptimal in vitro oocyte maturation and embryo culture conditions that compromise developmental competence. Consequently, future efforts must continue to optimize oocyte IVM, IVF, and extended culture protocols to maximize both the quantity and quality of blastocysts available for biopsy and genomic analysis [27].

Embryo biopsy serves as an essential step in genomic selection by bridging embryo culture and genetic testing, enabling pre-implantation genetic analysis. Its primary role is to provide sufficient DNA material for genotyping without significantly compromising embryonic developmental potential [28,29,30]. In this study, trophoblast cells from sheep blastocysts were biopsied using a microblade method [16]. Although cell counts were not individually verified, preliminary sampling indicated that ~75% of biopsies yielded 5–20 cells. Following 6 h of post-biopsy culture, the embryo recovery rate reached nearly 100%. The conception rate after fresh biopsied blastocyst transfer did not differ significantly from that of non-biopsied controls, indicating that the biopsy procedure itself did not impair embryonic developmental competence. However, a marked reduction (>30%) in the survival rate was observed after vitrification and thawing of biopsied embryos, suggesting increased cryosensitivity due to the biopsy. This phenomenon aligns with observations in human assisted reproduction, where biopsy techniques influence frozen-thawed embryo transfer outcomes [31,32,33]. To mitigate freezing-induced damage for biopsied blastocyst, future efforts could focus on refining biopsy techniques [34], such as adopting laser-assisted drilling, which minimizes zona pellucida disruption and may enhance cryotolerance. Additionally, optimizing cryopreservation protocols specifically tailored for biopsied embryos, including specialized freezing media and procedures, represents a critical direction for improving post-thaw survival and pregnancy rates [35,36].

Accurate genomic selection of pre-implantation embryos hinges on the recovery of sufficient DNA from the limited number of cells obtainable by trophectoderm biopsy [14]. WGA is therefore routinely employed to generate microgram quantities of DNA from picogram inputs [22,37]. In livestock genomics, the methods of WGA have also been widely explored, especially in the research of genomic selection in cattle embryos. The principal WGA platforms currently used in cattle embryo genotyping are MDA, MALBAC, and linear amplification via transposon insertion (LIANTI) [16]. In the present study, we systematically compared MDA and MALBAC for amplifying DNA derived from ovine embryo biopsies. Under identical cell input conditions, MDA consistently yielded higher amplified DNA concentrations, superior genotyping call rates, and lower mismatch rates than MALBAC. This result aligns with the predominant use of MDA in bovine biopsied embryo genotyping, confirming its technical advantages and cross-species applicability [19]. The input cell number is another important factor affecting the accuracy of SNP genotyping of biopsied cells [23]. In cattle studies, a biopsy standard of 5–10 trophectoderm cells is commonly adopted to balance genotyping reliability with minimal impact on embryo viability [19,20]. A clear positive correlation was observed between the number of sheep embryo biopsy cells and mean genotyping call rate; beyond ten cells, the call rate exceeded 93.71%. Le Bourhis et al. previously demonstrated that call rates exceeding 84.7% yield near-perfect concordance between GEBVs derived from embryo biopsies and those from the corresponding calves for both milk production and conformation traits [24]. Clara Slade Oliveira et al. provided the first estimates of pre-implantation prediction accuracy for 305-day milk yield in Gir cattle, by demonstrating that BovineHD imputation achieved superior accuracy (0.82) compared to Z-Chip imputation (0.55) or no imputation (0.62) [18]. This indicates that the genotyping system established for sheep embryos in this study meets the accuracy threshold required for practical breeding applications. Taking into account the damage caused by biopsy to the embryo and the impact of cell count on call rate we conclude that a biopsy comprising 10–20 trophectoderm cells represents the optimal compromise for embryo genomic selection in sheep, ensuring robust SNP genotyping while preserving the developmental competence of the biopsied embryo.

Low template input and inherent biases in WGA frequently introduce genotyping errors and depress call rates in embryonic biopsy samples, a problem that has been especially evident in bovine embryo genomic selection, where reported call rates range from 0.51 to 0.92 (mean 0.80) [18]. In the present study, ovine blastocyst biopsies amplified under our optimised WGA protocol achieved call rates of range from 0.86 to 0.98 (mean 0.94), indicating that we have obtained high-fidelity embryo biopsy cell genotyping data, which can also be reflected in the strong concordance between embryo GEBVs and the corresponding lamb offspring GEBVs with coefficient of determination for eight growth traits between 0.91 and 0.98. Nevertheless, we also further investigated the effect of genotype imputation on this concordance, as previous studies have shown that imputation can effectively improve the accuracy of embryonic genotypes, particularly for biopsy samples with low call rates. Imputation marginally increased concordance for five traits, whereas the remaining three were unchanged, presumably because the native call rate was already close to the technical ceiling. Collectively, these data indicate that our biopsy-to-genotype pipeline delivers ovine embryo GEBVs that are sufficiently robust for routine genomic selection without the need for post hoc imputation.

## 4. Materials and Methods

### 4.1. Animals and Sample Collection

Texel sheep were raised at Inner Mongolia Sino Sheep Technology Co., Ltd. (Ulanqab, China) under similar conditions, with free access to food, water, and natural lighting. From 60 donor ewes, 3348 cumulus-oocyte complexes (COCs) were obtained. The transfer of 160 biopsied blastocysts into recipient ewes resulted in 43 lambs, enabling genotyping analysis across all embryo-lamb pairs. Separately, to validate the WGA and imputation accuracy, 10 × WGS was performed on 39 embryo-derived DNA samples.

### 4.2. Laparoscopic Ovum Pick-Up (LOPU)

Texel donor ewes (Sino-Sheep Technology Co., Ulanqab, China) were managed according to an established superovulation protocol [38]. Estrus was synchronised in both donor and recipient ewes by inserting an intravaginal CIDR (InterAg, Hamilton, New Zealand) on any day of the cycle. Ten days after CIDR insertion, donors received six intramuscular injections of FSH (30 IU each; Sansheng Pharmaceutical, Ningbo, China) at 12-h intervals. LOPU was performed approximately 12 h after the final FSH injection (day 13). Under general anaesthesia, a 10 mm laparoscope was introduced through the ventral midline to visualise the ovaries. After exteriorisation and stabilisation with atraumatic grasping forceps, all follicles ≥3 mm in diameter were aspirated using a 20-G needle connected to continuous vacuum (15 mmHg) containing aspiration medium composed of TCM-199, 0.3% (*w*/*v*) bovine serum albumin (BSA, Sigma Chemicals Co., St. Louis, MO, USA) and 100 IU/mL penicillin.

### 4.3. Oocytes Collection and In Vitro Maturation (IVM)

The cumulus-oocyte complexes (COCs) were selected under stereomicroscope. Recovered COCs exhibiting normal morphology, homogeneous cytoplasm and ≥3 intact layers of compact cumulus cells were selected for in vitro embryo production. Selected COCs were washed three times in HEPES-buffered TCM-199 (Thermo Fisher Scientific, Shanghai, China) supplemented with 2% (*v*/*v*) fetal bovine serum (FBS) and matured in groups of 15–20 in 100 μL droplets of IVM medium covered with mineral oil at 38.5 °C, 5% CO_2_ and maximum humidity. IVM medium consisted of TCM-199 (Thermo Fisher Scientific, Shanghai, China) supplemented with 10 μg/mL FSH (Folltropin-V; Bioniche Animal Health, Belleville, ON, Canada), 10 μg/mL LH (Bioniche), 1 μg/mL 17β-estradiol, 100 μM DL-cysteamine, 10% (*v*/*v*) FBS and 1% (*v*/*v*) penicillin-streptomycin (Thermo Fisher Scientific, Shanghai, China). After 24 h IVM, oocytes were processed for in vitro fertilization.

### 4.4. In Vitro Fertilization and Embryo Culture

Following IVM, COCs were denuded by three washes in synthetic oviductal fluid (SOF; Caisson Laboratories, Rexburg, Idaho, USA) containing 0.2% (*w*/*v*) hyaluronidase and then transferred to the IVF medium (SOF supplemented with 2% oestrous sheep serum, 3 mg/mL BSA, 6 IU/mL heparin sodium and 50 IU/mL gentamicin). Frozen semen straws were thawed in a 39 °C water bath for 1 min. After dilution in pre-equilibrated IVF medium, spermatozoa were selected by a 30 min swim-up at 38.5 °C under 5% CO_2_ in humidified air. The upper motile fraction was collected, centrifuged at 200× *g* for 5 min, and the pellet resuspended in IVF medium to give a final concentration of 1 × 10^6^ spermatozoa/mL. Oocytes were co-incubated with spermatozoa for 20 h at 38.5 °C, 5% CO_2_, maximum humidity. Presumptive zygotes were washed three times in IVC medium and cultured in groups of 25–30 in 50 µL droplets of SOF supplemented with 1% (*v*/*v*) BME-essential amino acids, 1% (*v*/*v*) MEM-nonessential amino acids, 1 mM l-glutamine and 3 mg/mL BSA under 38.5 °C, 88% N_2_, 5% CO_2_ and 7% O_2_. Cleavage and blastocyst rates were recorded at 48 h and on day 6 post-IVF, respectively. The obtained blastocysts are used for embryo biopsy.

### 4.5. Embryo Transfer

Morphologically normal blastocysts were selected for transfer. Recipient ewes were fasted for ≥20 h, then subjected to laparoscopic transfer under general anaesthesia. Embryo transfer was performed as recommended by the International Embryo Technology Society [39]. On the day of embryo transfer the presence of a corpus luteum was determined via laparoscopy, and embryos were transferred ipsilateral to the corpus luteum. A 5 mm endoscope was introduced through the ventral midline to visualise the uterus; each blastocyst was loaded into a 0.25 mL straw and deposited into the lumen of the ipsilateral uterine horn via a 16-gauge transfer catheter. Pregnancy was diagnosed 40 days post-transfer by trans-abdominal ultrasonography.

### 4.6. Embryo Biopsy and Sample Preparation

Two hundred ovine blastocysts were biopsied manually under a Nikon Eclipse TS100 inverted microscope using sterile stainless-steel microblades. Embryos were placed in 35-mm dishes containing pre-equilibrated culture medium whose base had been lightly scratched to create traction; this eliminated the need for a holding pipette, as described by Bredbacka et al. [40]. With the blastocyst stabilized against a scratch, a microblade was gently pressed against the trophectoderm and moved in a slow, oscillating motion until 10–20 trophectoderm cells were cleanly excised. The biopsied cells was aspirated with a finely drawn pipette, washed three times in 0.1% (*w*/*v*) PVA-PBS, and transferred to 8 µL lysis buffer containing 20 mg/mL proteinase K (Invitrogen, Carlsbad, CA, USA) for downstream analysis. For cell number-based experiments, embryos was subjected to zona pellucida removal using 5% pronase for 1–3 min, followed by digestion in 300 µL of enzyme solution (Trypsin-EDTA:Accutase = 1:1) at 38 °C for 15–30 min. The cells were dissociated in 100 µL PBS-PVA via pipetting, washed three times, and aliquoted into 200 µL tubes containing 4/8 µL lysis buffer for WGA.

### 4.7. Whole-Genome Amplification

Whole-genome amplification (WGA) was performed using two distinct methods: Multiple Annealing and Looping-Based Amplification Cycles (MALBAC) and Multiple Displacement Amplification (MDA). Amplification of collected biopsied blastocyst via the MDA method was carried out using the REPLI-g Single Cell Kit (Qiagen, Hilden, Germany) according to the manufacturer’s standard protocols. For comparison, parallel samples were amplified using the MALBAC method with Single Cell WGA Kit (Yikon Genomics, Shanghai, China). All amplification procedures were conducted in a PCR thermocycler.

In addition, genomic DNA from jugular venous blood samples of one-month-old lambs was extracted using the Blood Genomic DNA Extraction Kit (TIANGEN, DP348) in accordance with the manufacturer’s instructions.

### 4.8. DNA Quantification and Quality Control

DNA concentrations and purity (based on the OD260/280 ratio) was measured using a NanoDrop-2000 spectrophotometer (Thermo Fisher Scientific, Wilmington, DC, USA). A subset of samples was diluted with DEPC-treated water to concentrations of 50 ng/µL, 200 ng/µL, and 2000 ng/µL.

### 4.9. SNP Chip-Based Genotyping

Genotyping was performed using the Illumina Infinium OvineSNP50K BeadChip v3 on both amplified embryo-derived DNA and venous blood DNA samples. Genotyping was performed using the Illumina Infinium OvineSNP50K BeadChip v3. The chip provides genome-wide coverage of ovine variation and enables high-throughput genotyping. Both amplified embryonic cell DNA and venous blood DNA samples were processed. Services were provided by Beijing Compass Biotechnology Co., Ltd. (Beijing, China). Raw data were aligned to the Oar v4.0 reference genome (GCF_000298735.2) using GenomeStudio 2.0 (Illumina, San Diego, CA, USA)with standard cluster files. Consistency rate and mismatch rate between each sample were calculated using a manually compiled Python 3.9 script to compute genotype concordance per sample and per SNP.

### 4.10. Whole-Genome Sequencing and Imputation

MDA-amplified samples were used to construct libraries with approximately 500 ng of WGA product and subsequently subjected to 10× whole-genome sequencing on the DNBSEQ-T7 platform (MGI Tech. Co., Ltd., Shenzhen, China), with sequencing services provided by Glbizzia Biosciences Co., Ltd. Raw sequencing reads were aligned to the Oar v4.0 reference using BWA-MEM. Duplicate reads were marked with Picard, and base quality recalibration and variant calling were performed using GATK Best Practices. Initial variant calls were generated with GATK HaplotypeCaller and merged into a cohort VCF. Quality control was performed using PLINK 1.9 with the following criteria(—maf 0.05, —hwe 1 × 10^−5^, —mind 0.1, and —geno 0.1): minor allele frequency (MAF) ≥ 0.05, Hardy–Weinberg equilibrium (HWE) *p*-value ≥ 1 × 10^−5^, and individual and SNP call rates ≥90%. Common SNPs between WGS data and the OvineSNP50K chip were retained for imputation training, resulting in 53,685 valid SNPs. Identity-by-descent (IBD) analysis was conducted using PLINK 1.9 to confirm sample relatedness and construct a reference panel. Imputation was carried out with Beagle5.4. Performance was assessed through the change in call rate, post-imputation genotype concordance, rescue rate (percentage of successfully imputed missing genotypes), and correction rate (percentage of erroneous chip genotypes corrected to the true genotype via imputation).

### 4.11. Genomic Prediction and GEBV Calculation

Genotypes from 43 embryo-lamb pairs were analyzed. GEBVs for eight growth traits: birth weight (BW), body weight at 90, 180 and 240 days, and average daily gain during these intervals, were estimated using the BLUP90 family of programs (accessed on 23 September 2024) based on a reference population of 971 Australian White sheep. The GBLUP model was:

(1) y=1b+Za+e,

where  y is the vector of phenotypic observations, b is the vector of fixed effects, a is the vector of random additive genetic effects with $a\sim N(0,G\sigma_{a}^{2}),e$ is residual erroris the residual vector with $e\sim N(0,I\sigma_{e}^{2})$, and Z are design matrices. The genomic relationship matrix G was constructed using VanRaden’s method [3]:

(2) $$G=\frac{M\prime M}{\sum 2p_{i}(1-p_{i})}$$

where M is the genotype matrix with entries coded as $g-{2p}_{i}$ for SNP genotype g, and $p_{i}$ is the minor allele frequency of the i-th SNP.

Genomic reliability was calculated as:

(3) $$Reliability=1-\frac{PEV}{\sigma_{a}^{2}}$$

where PEV is the prediction error variance.

### 4.12. Technical Replicate and Batch Effect Management

To ensure the technical robustness of genotyping data from low-input embryonic samples, a multi-layered quality control strategy was implemented. For each WGA reaction, success was determined by measuring DNA concentration using a NanoDrop-2000 spectrophotometer (Thermo Fisher Scientific, Wilmington, DC, USA) and confirming the presence of a dominant high-molecular-weight DNA band via agarose gel electrophoresis. Only samples passing these criteria were submitted for genotyping. To minimize technical batch effects, all MDA-based WGA kits and Illumina OvineSNP50K BeadChips were pre-ordered from the same manufacturing lots. Furthermore, samples were randomized across genotyping chips, and sub-experiments were conducted within single batches where possible.

### 4.13. Statistical Analysis

Data were analyzed using GraphPad Prism v10 and IBM SPSS Statistics v22 software. All collected data were analyzed using descriptive statistics. Student’s *t*-test was employed to compare blastocoel re-expansion rates at different recovery time points (e.g., 4 h vs. 6 h) and to evaluate the effect of lysis buffer volume on DNA concentration, call rate, and mismatch rate. Two-way analysis of variance (ANOVA) was applied to assess the main effects and interaction between amplification method (e.g., MDA vs. MALBAC) and cell number on DNA concentration, call rate, and mismatch rate. One-way ANOVA was still used to examine the influence of DNA loading concentration on these outcome variables. A significance threshold of *p* < 0.05 was considered statistically significant, with *p* < 0.001 indicating high significance.

## 5. Conclusions

This study systematically evaluated whole-genome amplification and 50K SNP chip genotyping performance for ovine embryo biopsy samples containing varying numbers of trophectoderm cells (1, 5, 10, 20, 30). The results indicated that amplifying 10-cell samples via the MDA method yielded optimal DNA quantity (2179 ± 35.83 ng/μL), along with a high SNP call rate (93.71 ± 0.90%) and low genotyping mismatch rate (2.40 ± 1.11%), demonstrating that approximately 10 cells per embryo are sufficient for reliable genomic evaluation. Furthermore, using 8 μL of lysis buffer significantly improved amplification stability. A robust genotyping workflow was established based on 10–15 biopsied cells. The initial chip call rate ranged from 86.01% to 98.21%, which increased to 97.63–98.40% following imputation using whole-genome sequencing data. GEBVs for eight traits showed strong concordance (R^2^ = 0.912 − 0.975) between embryos and their resulting lambs. Imputation improved predictive accuracy for five of these traits.

In summary, this study developed and optimized a high-accuracy genomic analysis system for minute quantities of embryonic cells in sheep, enabling early-stage genetic evaluation. This approach provides a reliable technical foundation for elite sheep breeding, significantly shortens generation intervals, and accelerates genetic progress.

## Data Availability

The original contributions presented in this study are included in the article. Further inquiries can be directed to the corresponding authors.

## Supplementary Materials

The following supporting information can be downloaded at: https://www.mdpi.com/article/10.3390/ijms26199738/s1.

## Abbreviations

The following abbreviations are used in this manuscript:

GS	Genomic selection
MDA	Multiple Displacement Amplification
MALBAC	Multiple Annealing and Looping Based Amplification Cycles
WGA	Whole-genome amplification
GEBV	Genomic estimated breeding value
LOPU	Laparoscopic Ovum Pick-up
IVM	In vitro maturation
IVF	In vitro fertilization
IBD	Identity-by-Descent
SNP	Single nucleotide polymorphism
IVEP	In vitro embryo production
OPU	Ovum pick-up
LIANTI	Linear amplification via transposon insertion
CIDR	Controlled internal drug release
COCs	Cumulus-oocyte complexes
GBLUP	Genomic best linear unbiased prediction
