# Supplementary material for: The Establishment of a Sheep Embryo Genomic Selection System
Source: Int J Mol Sci. 2025 Oct 7;26(19):9738. doi: 10.3390/ijms26199738 (PMC12525284; doi:10.3390/ijms26199738)
Supplement: Supplementary file 1 [file ijms-26-09738-s001.zip › ijms-3896350-supplementary.pdf]

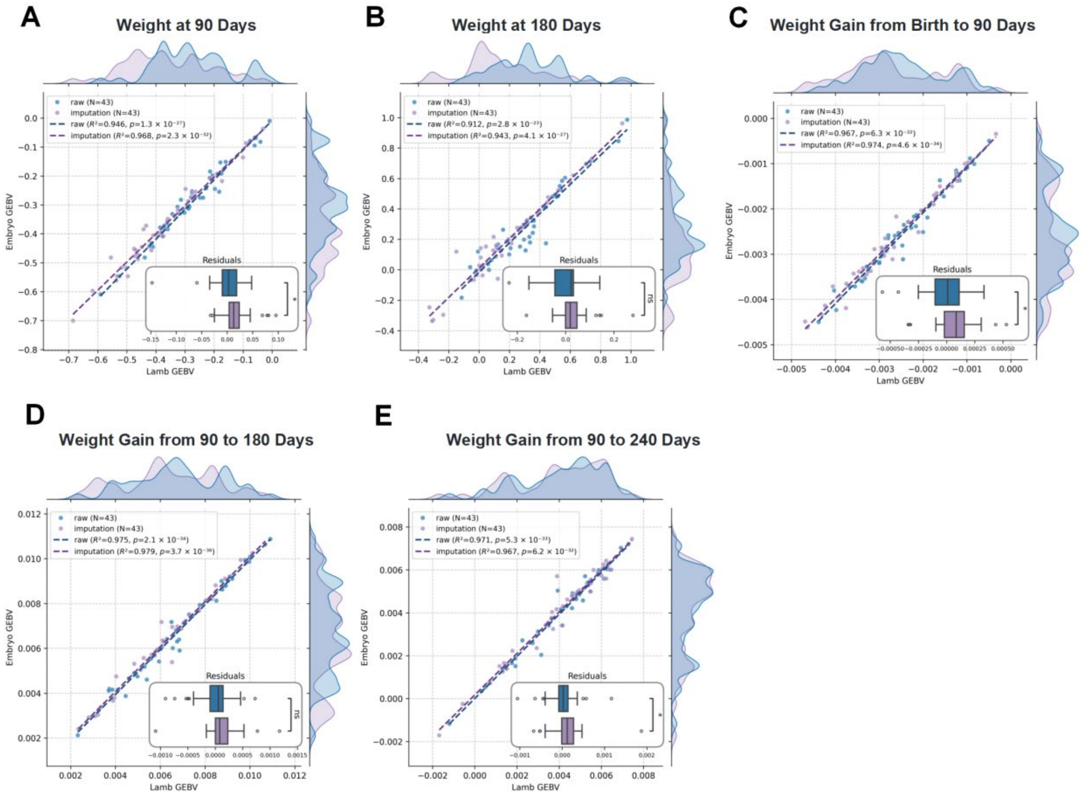

Figure S1. Embryo-lamb GEBV regression-residual composite plot: linear regression of GEBVs derived from embryo genotypes (y-axis) against lamb GEBVs (x-axis) is presented for both raw (blue) and imputed (gray purple) genotypes (n = 43). (A) weight at 90 days, (B) weight at 180 days, (C) weight gain from birth to 90 days, (D) weight gain from 90 to 180 days, and (E) weight gain from 180 to 240 days. The lower panel shows corresponding residuals. Dashed lines represent fitted regressions.
